# Supplementary material for: Intramedullary Spinal Cord Tumors: Whole-Genome Sequencing to Assist Management and Prognosis
Source: Cancers (Basel). 2024 Jan 18;16(2):404. doi: 10.3390/cancers16020404 (PMC10814932; doi:10.3390/cancers16020404)
Supplement: Supplementary file 1 [file cancers-16-00404-s001.zip › cancers-2727798-supplementary.pdf]

cancers

Supplementary Materials: Intramedullary Spinal Cord Tumors: Whole Genome Sequencing to Assist Management and Prognosis

Miguel Mayol del Valle [1,*], Bryan Morales [2], Brandon Philbrick [3], Segun Adeagbo [3], Subir Goyal [4], Sarah Newman [1], Natasha L. Frontera [5], Edjah Nduom [1], Jeffrey Olson [1], Stewart Neill [2] and Kimberly Hoang [1]

1 Emory University Hospital, Department of Neurosurgery, 1365 Clifton Road NE, Suite B6200, Atlanta, GA 30322 USA
2 Emory University Hospital, Department of Neuropathology, 1364 Clifton Road, NE Room H-184, Atlanta, GA 30322 USA
3 Emory University School of Medicine, 100 Woodruff Circle, Atlanta, GA 30322 USA
4 Biostatistics Shared Resource Department, Winship Cancer Institute of Emory University, 1365-C Clifton Road, NE, Atlanta, GA 30322 USA
5 University of Puerto Rico Medical Sciences Campus, School of Medicine, PO Box 365067, San Juan, PR 00936-5067
* Correspondence: mmayold@emory.edu

Table S1. (Patients with genetic testing).

Age	Sex	BMI	Comorb	Location	Resect %	PO KPS	Hosp (days)	Diagnosis	Important Mutations	Gain	Loss	LOH	Total	Radiation (Dose)	Chemo	Progre	Follow-up (Days)
68	Female	21.9	HTN	Thoracic	100%	60	9	LGA (WHO II)	IDH WT	n/a	n/a	n/a	n/a				8
70	Female	26.9	DM	Cervical	78-99%	40	16	Ependymoma (WHO II)	IDH WT	n/a	n/a	n/a	n/a			YES-Surgery	754
38	Male	18		Thoracic	Biopsy	50	8	LGA (WHO II)	IDH WT, BRAFV600E	n/a	n/a	n/a	n/a	Fractionated (50.4)			303
37	Female	22.3		Cervical	100%	90	5	Ependymoma (WHO II)	IDH WT	n/a	n/a	n/a	n/a				36
71	Female	44.4	HTN, CAD, DM	Thoracic	0%	60	8	Astrocytoma (WHO III)	IDH WT, P53 overexp	n/a	n/a	n/a	n/a	Fractionated (50.4)		YES-Radiation	223
47	Female	38.1		Cervical	100%	80	4	LGA (WHO II)		n/a	n/a	n/a	n/a				359
61	Female	22.5		Cervicothor	78-99%	40	13	Ependymoma (WHO II)		0	2	0	2				107
55	Female	28.3		Cervical	78-99%	60	7	Ependymoma (WHO II)		0	2	0	2	Fractionated Proton (50.4)			610
54	Male	28.3		Cervical	Biopsy	80	3	Ependymoma (WHO II)	IDH WT	5	3	0	8	Fractionated Proton (52.2)			467
61	Male	28.2	HTN, MI	Cervical	78-99%	60	21	Ependymoma (WHO II)		2	6	1	9			Yes-Death	43
29	Male	34.3	HTN, DM	Thoracic	78-99%	80	4	Glioblastoma (WHO IV))	IDH WT, ATRX Del, MGMT Meth, KRAS pos	6	6	1	13	Fractionated (48.6)	Temodar	YES-Chemo/Rad	143
68	Female	24.9		Lumbar	100%	80	5	Myxopapillary Ependymoma (WHO II)		6	10	0	16				536
41	Female	38.3	HTN, CAD	Cervical	<50%	70	11	Glioblastoma (WHO IV))	IDH WT, ATRX Del, MGMT Meth, P53 overexp	0	25	5	30			Yes-Hospice	39
54	Male	23.5		Cervical	100%	90	6	Ependymoma (WHO II)		37	0	0	37				161
